# Modeling Conformational Ensembles of Slow Functional Motions in Pin1-WW

**DOI:** 10.1371/journal.pcbi.1001015

**Published:** 2010-12-02

**Authors:** Faruck Morcos, Santanu Chatterjee, Christopher L. McClendon, Paul R. Brenner, Roberto López-Rendón, John Zintsmaster, Maria Ercsey-Ravasz, Christopher R. Sweet, Matthew P. Jacobson, Jeffrey W. Peng, Jesús A. Izaguirre

**Affiliations:** 1 Interdisciplinary Center for Network Science and Applications, Notre Dame, Indiana, United States of America; 2 Department of Computer Science and Engineering, University of Notre Dame, Notre Dame, Indiana, United States of America; 3 Graduate Group in Biophysics and Department of Pharmaceutical Chemistry, University of California, San Francisco, San Francisco, California, United States of America; 4 Center for Research Computing, University of Notre Dame, Notre Dame, Indiana, United States of America; 5 Facultad de Ciencias, Universidad Autónoma del Estado de México, Toluca, México; 6 Department of Chemistry and Biochemistry, University of Notre Dame, Notre Dame, Indiana, United States of America; 7 Department of Physics, University of Notre Dame, Notre Dame, Indiana, United States of America; National Cancer Institute, United States of America and Tel Aviv University, Israel

## Abstract

Protein-protein interactions are often mediated by flexible loops that experience conformational dynamics on the microsecond to millisecond time scales. NMR relaxation studies can map these dynamics. However, defining the network of inter-converting conformers that underlie the relaxation data remains generally challenging. Here, we combine NMR relaxation experiments with simulation to visualize networks of inter-converting conformers. We demonstrate our approach with the apo Pin1-WW domain, for which NMR has revealed conformational dynamics of a flexible loop in the millisecond range. We sample and cluster the free energy landscape using Markov State Models (MSM) with major and minor exchange states with high correlation with the NMR relaxation data and low NOE violations. These MSM are hierarchical ensembles of slowly interconverting, metastable macrostates and rapidly interconverting microstates. We found a low population state that consists primarily of holo-like conformations and is a “hub” visited by most pathways between macrostates. These results suggest that conformational equilibria between holo-like and alternative conformers pre-exist in the intrinsic dynamics of apo Pin1-WW. Analysis using MutInf, a mutual information method for quantifying correlated motions, reveals that WW dynamics not only play a role in substrate recognition, but also may help couple the substrate binding site on the WW domain to the one on the catalytic domain. Our work represents an important step towards building networks of inter-converting conformational states and is generally applicable.

## Author Summary

Proteins in their native state can adopt a plethora of shapes, or conformations; this conformational plasticity is critical for regulation and function in many systems. However, it has remained difficult to determine what these different conformations look like at the atomic level. We present a novel way to use Nuclear Magnetic Resonance, Molecular Dynamics Simulations, and Markov State Models to uncover a map of this plethora of conformations that is consistent with the available data. We applied this method to study the intrinsic dynamics used in substrate binding by the WW domain of the Pin1 proline cis-trans isomerase and found that the NMR data were best explained by two slowly-interconverting sets of many metastable conformations rather than two distinct macrostates. Substantial value is added to the NMR data by our method since it provides a kinetic “map” of conformational changes consistent with the observed relaxation data. Such an approach, in combination with information theory, helped us to identify specific conformational changes that might couple substrate binding at the Pin1 WW domain to the catalytic subunit.

## Introduction

Protein-protein interactions are often mediated by flexible motifs or domains that make conformational transitions on slow 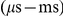 time scales. Flexibility helps accommodate the versatile binding properties of interaction domains [1]. Nuclear Magnetic Resonance (NMR) relaxation experiments have emerged as a premier tool for revealing the location and time scale of these transitions [2], [3]. More recently, Kay and co-workers [4] have shown that for the case of transitions between two states, NMR relaxation dispersion can provide structural models of the minor populated state, which is not directly observable. Also, NMR methods to detect correlated motion are increasing [5],[6], yet remain technically challenging.

The number of NMR observables will generally only be a subset of the total degrees of freedom of the system. To maximize data interpretation, it is reasonable to turn to molecular dynamics simulations. Such simulations can retain all molecular degrees of freedom, and offer conformational ensembles that may be evaluated on the basis of their consistency with experiment. Current NMR spin relaxation experiments can reveal microsecond-millisecond conformational dynamics in proteins on a residue-by-residue basis [7], [8]. Here, we explore the potential for the clustering of molecular dynamics simulations to capture such motions.

Pioneering computational studies to identify the conformations probed by NMR relaxation dispersion include work by Ernst *et al.* [9] and Palmer *et al.* [10]. In the latter study, analyses of chemical shift and structural databases with SHIFTX enabled modeling of a minor state conformation. Correlated protein motions were studied by comparing MD simulations with NMR relaxation and further NMR spectroscopic motions [11]. Extensive Residual Dipolar Couplings (RDC) measurements from multiple alignment media can aid in calculating an ensemble of conformations consistent with the experimental data that accounts for slow motions over a broad timescale [12], [13], and are quite complementary to relaxation data. For instance, de Groot *et al.* were able to identify all known conformations of ubiquitin from an RDC-derived ensemble [13]. Related work by Markwick, McCammon, Blackledge and collaborators, has correlated RDC to long Accelerated Molecular Dynamics (AMD) simulations [14].

Since resolving millisecond dynamics through very long explicit-solvent MD is not feasible at the present time, we instead use more efficient ways of generating a kinetic model. Markov State Models (MSM) are kinetic graph models with 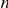 nodes representing metastable, or long lived states that partition configuration space, and edges representing transition probabilities among states. MSM directly incorporate heterogeneity of pathways in protein dynamics, and allow “parallelization” of the kinetic estimation by breaking the problem of estimating conformational transitions. MSM can be built by simulating an ensemble of MD simulations out of multiple metastable states. Recent work has shown quantitatively the advantage of constructing equilibrium ensembles by starting relatively short simulations from different starting points in configuration space [15]–[17]. To be able to estimate transition probabilities it is important that these simulations preserve dynamical information, even though one can use Monte Carlo schemes such as replica exchange (REMD) to identify some initial, putative states from which to shoot MD simulations. An attempt at creating a Markov model from REMD using the ansatz that kinetic transitions are allowed (guessed) between states that have sufficient structural similarity has been used to study protein folding pathways [18].

Specifically, we construct a hierarchical representation of the free energy landscape by clustering Markov State Models into exchange states that correlate well with the NMR experiments. The correlation between states from simulation and NMR relaxation experiments is achieved through chemical shift computations. These states form an ensemble of inter-converting metastable macrostates and rapidly converting microstates. Critically, since the simulations are unrestrained, they enable a nearly unbiased identification of metastable states, their interconversions, and their populations. Furthermore, the correlation with NMR provides a novel way of determining important parameters of the MSM, such as the number of macrostates needed.

We illustrate our approach on the conformational dynamics of the Pin1-WW domain. WW domains are a family of modular recognition domains that mediate protein-protein interactions in cell signaling networks. These compact domains (38–40 residues) contain two conserved tryptophans (W) spaced approximately 20 residues apart. They function as interaction domains of polyproline II helix motifs on the surfaces of other proteins [19]. WW domains are recruited by numerous cell signaling proteins implicated in cancer, Alzheimer's disease, Huntington's disease, muscular dystrophy, and Liddle's Syndrome hypertension [20].

**Figure 1:**
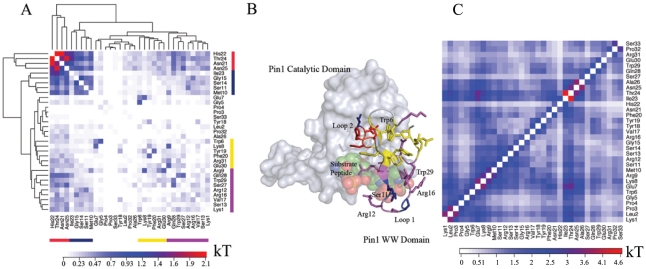
Correlated motions couple the catalytic domain interface to the substrate-binding loop of Pin1's WW domain. The WW domain is shown in cartoon and sticks, the catalytic domain as a surface, and the substrate in spheres. The structure shown is from PDB entry 1F8A. Only the WW domain was simulated; the catalytic domain is only shown for reference. (A) Hierarchical clustering of the mutual information between residues' torsions identifies several functionally important groups of residues. (B) Most residues in the red cluster lie in the catalytic domain interface and are correlated with residues in magenta cluster, which includes a number of key substrate-binding residues. All residues exhibiting slow motions in NMR experiments are in either the red or magenta clusters. (C) Mutual information between 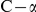 atoms complements torsional analysis and importantly captures correlated motions of secondary structure elements, highlighting correlated motions between the first 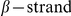 (residues 7–9) and Loop 1 (residues 10–16), between the first 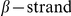 and the second 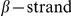 (residues 17–21), and between the C-terminal part of Loop 2 and the beginning of the third 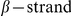 (residues 23–26) and the rest of the protein.

Figure 1B shows the structure of the Pin1-WW domain. WW domains share a common three-stranded 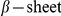 architecture; yet, they display different binding preferences, which have been attributed to the sequence variability of a flexible binding loop between 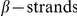 1 and 2 (Loop 1, residues 11 to 16 according to sequence numbering of PDB 1i6c). This has motivated numerous studies of the Loop 1 to better understand its biophysical properties [21]. Recently, Peng *et al.* investigated the backbone NH dynamics of Pin1-WW, using a variety of 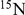 NMR spin relaxation experiments [22]. The result was a residue-by-residue profile (33 NH bonds) of bond motion that highlighted Loop 1 residues as sites undergoing significant microsecond-millisecond dynamics. Notably, 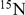 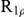 dispersion experiments of Arg-12 in Loop 1 revealed intrinsic conformational dynamics on millisecond time scale, which decreased upon phosphopeptide ligand binding. Moreover, mutating Loop 1 simultaneously changed Pin1-WW binding affinity and dynamics. This suggested that the Loop 1 sequence encodes motions critical for complex formation.

In this work we consider 2 ensembles to map conformational transitions: 55,490 short MD simulations started from configurations obtained from a 554 ns long trajectory of apo Pin1-WW domain (PDB 1i6c) in explicit solvent (“Extended 1”), and 250 longer MD simulations (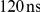 in average) started from random configurations out of the same original long simulation (“Extended 2”). From these simulations we construct a Markov State Model (MSM) with 1,000 rapidly converting microstates and 40 metastable macrostates. We further cluster the MSM macrostates to build major and minor exchange states that correlate very well with NMR 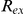 values, which measures the excess transverse relaxation arising from conformational exchange. Chemical shift calculations are used to compute correlation to 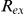. H-bond network information is used to guide the clustering of macrostates into exchange states (see Methods).

Analysis of these MSM using network theory reveals the presence of basins visited by most pathways among macrostates, i.e., “kinetic hubs”. The main kinetic hub consists primarily of holo-like structures, close to the structure of Pin1-WW bound to CDC25 (PDB 1i8g). This suggests the pre-existence of conformational equilibria in the intrinsic dynamics of the apo Pin1-WW, which includes slow transitions between apo versus holo conformations. There is no a priori reason why this should be so: the slow motion could have been between 2 states that are not competent to bind substrate. This lends further credence to the idea that intrinsic, slow protein dynamics are often functionally relevant [13], [23]. While MSM have also been used for analysis of folding pathways for Pin1-WW [17], to the best of our knowledge they have not been used for the study of intrinsic, functional dynamics of Pin1-WW.

Finally, we analyze simulation data using a thermodynamics-based mutual information metric to find pairs of residues with correlated conformations in the conformational ensemble. In a conformational ensemble, it does not matter whether one residue moves, then another, so we can use correlated conformations and correlated motions interchangeably, as no time offsets are used. This approach provides an analysis of correlated motions that is complementary to NMR 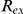 measurements. We find that Loop 1 residues form a cluster that is correlated with key residues that lie in the catalytic domain interface. These correlations are mediated by some residues in the 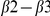 loop (Loop 2), providing mechanistic insight into how Loop 1 dynamics may affect function of Pin1.

## Methods

### Nuclear Magnetic Resonance

The motions of the backbone NH bonds of Pin1-WW at 278K were previously characterized via Lipari-Szabo “model-free” analyses [24] of 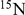 relaxation parameters. NH bonds experiencing slow 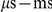 dynamics were those yielding significant 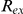 values after the Lipari-Szabo analyses. 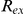 is the excess transverse relaxation caused by modulation of the 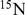 chemical shift that results from underlying 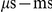 dynamics.

Arg-12, Ser-13, and Gly-15 gave large 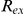 with Arg-12 showing the largest contribution (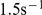). These 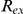 values did not represent the full extent of exchange-broadening, due to the narrow inter-pulse delays in the 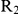 Carr-Purcell-Meiboom-Gill (CPMG) experiments. Later, complementary 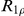 relaxation dispersion measurements [3] on resonance with Arg-12, showed that the Arg-12 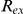 reflected a two-state dynamic process involving major and minor states, with a minor state population 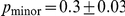, a net exchange rate constant 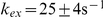, and a chemical shift difference of 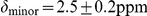 between the two putative states [22].

An approximate expression relating 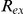 to chemical shift difference and exchange rate constant for fast exchange and on resonance with the major species is [3]:
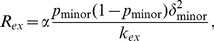
(1)where 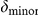 is the chemical shift difference between the major versus minor state, 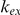 is the two-state rate constant, and 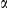 is a fraction that accounts for the partial quenching of exchange during the CPMG spin-lock. While the above 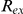 expression is only approximate, it is sufficient to define the relative extent of exchange along the protein sequence.

The experimental NMR data were well fit by established two-state models [3],[25] and the number of NMR observables did not justify more complex models. Generally, based on relaxation data alone, the inadequacy of two-state exchange scenarios can be difficult to assess, unless the constituent exchange processes have highly divergent time-scales. Since 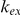 and 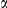 cannot currently be obtained from the simulations, we only look for correlations of the relative 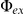 of each residue with respect to the 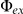 for Arg-12. The 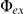 estimator is computed as
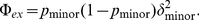
(2)We investigated different ways of clustering results from MD simulations to obtain definitions of major and minor exchange states such that
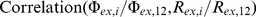
(3)is maximized. The chemical shift difference 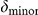 in Eq. (2) is estimated as the difference of the chemical shift means for the major and minor state. The chemical shift for each conformation is estimated using SHIFTX [26], as described below.

### Molecular Dynamics Simulation

Simulation of human Pin1 WW domain (PDB code : 1I6C) has been carried out with the CHARMM 27 force field using NAMD 2.6 [27]. The peptide has a total of 39 residues. Since we are interested in long time scale dynamics of Loop 1 as well as on being able to match the experimental setup, we removed 6 residues from the C-terminus (residues 34–39). The peptide is solvated with TIP3P water. We added a layer of 7Å water around the protein. The solvated system has 4,958 atoms. The size of the periodic box containing the system is 36.4Å/40.1Å/33.7Å. The system was minimized and equilibrated using NAMD 2.6. We equilibrated the system using NPT ensemble until no significant change in potential energy or RMSD was observed.

After equilibration, we ran simulation in canonical ensemble (NVT) for 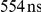. We used a Langevin thermostat for temperature control. A cutoff of 12Å was used for calculating non-bonded interactions. A 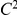 switching function for Lennard-Jones was applied starting at 10Å. Pairlist calculations were done at 14Å every 10 steps. We used SHAKE to constrain bonds in water molecules as well as the bonds to hydrogen in the peptide. A time step of 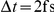 was used for updating the positions and velocities. Bonded and short-range non-bonded interactions were calculated once every 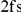. Long-range electrostatic interactions using particle mesh Ewald (PME) were calculated once every 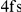, using an 1Å grid spacing. We ran the simulation at target temperature 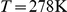 to match the experimental setup. We used a coupling constant of 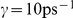 for heavy atoms. We recorded the system coordinates every 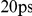 during the simulation.

From this trajectory, called 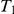, we obtained 27,745 frames. Analysis of 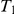 indicated that more sampling was required for proper identification of major and minor states of the system and to correlate with NMR 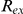 (see below). One way to solve this problem would be to run many microsecond trajectories with same starting position but different initial velocities. However, generating many microsecond trajectories requires a very long time.

#### Extended 1

We used a different simulation protocol to enhance sampling while exploiting parallelism. Let us assume that 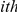 frame from trajectory 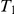 is 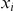. Enhanced local sampling around 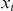 can be achieved by running 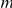 (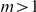 is an integer and finite) simulations 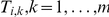 starting from 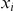 with different initial velocities. We ran each of these simulations for 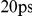. We stored system positions at every 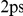. One advantage is that each 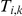 is independent and can run concurrently with each other. We collected 1,105,760 samples of the system, each 2 ps apart, that we call *Extended 1*.

#### Extended 2

We performed local sampling with fewer but longer trajectories. We picked a set of 125 conformations randomly from the set of all 27,745 frames. We ran 2 trajectories with different initial velocities from each of these 125 frames. On average, we ran each of these trajectories for 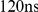. We stored the system positions once every 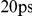. We collected 1,513,394 samples of the system, that we call *Extended 2*. Note that the latter's aggregate sampling is approximately 15 times the length of *Extended 1*'s.

### Chemical Shift Calculation

Chemical shift values for all the residues in the MD simulations are needed for estimating 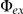, and are calculated using SHIFTX [26]. This software predicts chemical shifts from atomic coordinate data using classical equations that take into account ring currents, H-bonds and electric fields as well as hypersurfaces obtained from databases of observed chemical shifts. SHIFTX receives as input PDB coordinates or DCD trajectories and estimates the 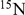 chemical shift of atoms in the side chains or backbone. We used SHIFTX to get diamagnetic chemical shift values for each residue of Pin1-WW domain for all frames from *Extended 1* and *Extended 2* simulation datasets.

### Markov State Model Construction

We built Markov State Models (MSM) out of the simulation data using the MSMBuilder package [28]. In this approach, one needs a criterion for clustering into microstates. Based on the evidence from NMR relaxation and structures of apo and holo WW, we focused on Loop 1 conformations to define different microstates. The rationale is that the 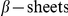 are relatively rigid and do not greatly contribute to conformational plasticity of intrinsic apo dynamics. We assume that Loop 1 conformations within a 3Å RMSD can interchange rapidly and thus are justified to belong to the same microstate. The approximate k-centers algorithm is used to create clusters of equal volume. In this work, it was enough to use 1,000 microstates to obtain a spread of 3Å on average.

The microstates are then furthered clustered into kinetically related states called macrostates. Using a transition probability matrix between microstates, for varying time lags, an MSM is constructed. The Perron Cluster Cluster Analysis (PCCA) uses the eigenvalues and eigenvectors of the microstate Markov state models to determine common kinetic features between microstates and cluster them in related groups. Finally, simulated annealing is used to maximize metastability and refine the macrostates obtained by PCCA. Metastability is the probability of staying in the same state after a lag time. We built MSM for varying numbers of macrostates, from 2 up to 40. One novel feature of our approach is that we used we used the correlation to 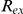 as explained below to determine the number of macrostates needed.

We constructed an initial MSM using the undersampled dataset Extended 1 and then we added the long trajectory data to this MSM, as suggested in [28], [29]. The MSM model was validated using standard methodologies, primarily by searching for a stationary distribution (Chapman-Kolgomorov test) and by looking at intrinsic time scales, cf. [30]. Some of the implied time scales for the MSM using 40 macrostates are shown on Figure S1. They present a Markovian behavior within statistical error for the slowest time scales. For the case of the WW transition matrix 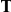, the stationary distribution 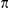 (see Figure S2) contains two macrostates of higher probability (numbers 9 and 38) than the rest of the macrostates. One interesting observation is that one of these *attractor-like* states (Macrostate 9) is an intermediate state, in RMSD sense, with respect to holo and apo structures. The transition matrix is given as supplementary Dataset S1 for the *Extended 1* dataset and as supplementary Dataset S2 for the *Extended 2* dataset.

Note that the free energy basin of Pin1-WW, as that of most proteins, is hierarchical, and thus there are many possible numbers of states for an MSM that can be chosen. However, it is important to note that only using 2 macrostates for the MSM gave a low correlation to the NMR 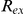, suggesting that using metastability alone may not be optimal for correlation to experiments.

### Conformational Exchange State Identification

We aim to identify major and minor exchange states from the simulation that maximize Eq. (3), and hence provide a connection to the NMR relaxation. We investigate three ways of identifying major and minor states: (1) Use of hydrogen bonding information; (2) clustering into 1,000 microstates and 2 macrostates of an MSM; (3) A hybrid method that uses chemical shift and hydrogen bonding information to accomplish further hierarchical clustering of the MSM, with 1,000 microstates, 40 macrostates, and 2 exchange states.

#### Method 1

Hydrogen bond (H-bond) reorganization has been proposed to affect protein slow dynamics, since correlated motions can be propagated through H-bond networks. This has been supported by the identification of a correlated polar network connecting ligand binding sites in interleukin-2 [31], and by studies of interstrand H-bonds in protein G [5] and loop H-bonds in a WW domain [22]. Based on this evidence, we hypothesize that H-bonds that are present in the Loop 1 of Pin1-WW are intrinsically related to the slow conformational exchange observed in NMR relaxation experiments.

We use the definition in Eq. (2) in a state search algorithm that aims to maximize the correlation between the calculated loop 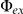 and the experimental 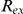, according to Eq. (3). We define a correlation function 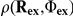 as the Pearson correlation coefficient between vectors 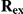 and 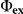 where 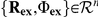 with 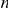 as the number of residues in the WW domain Loop 1. Furthermore, 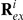 and 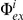 for 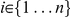 represent the 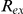 and 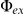 parameters for each residue in the Loop 1 of the WW domain, respectively. We can state our maximization problem as:
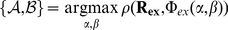
(4)where 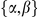 are the sets of trajectory frames with corresponding H-bonds in both, the state with smaller population (minor) and the one with the largest population (major), states that produce the estimated 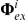 for each residue 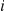. Using the chemical shift calculations and hydrogen bonds as described in the previous section (see Table S2 for a list of H-bonds found for Pin1-WW domain), Algorithm S1 is used to obtain the states that maximize the correlation function 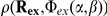.

The output of this method is a partition of 2 sets of conformations (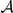 and 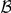) in the simulated trajectory for which we are able to compute chemical shift values. These sets represent an ensemble of domain coordinates giving rise to both the minor and major species in the 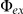 calculation. In this context, 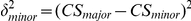, where 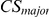 and 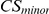 are the mean chemical shift values for all the frames present in 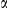 and 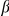. A fair correlation can be obtained (See Results).

#### Method 2

The conformations identified using H-bond information have no explicit relation to the free energy landscape. Thus, it is natural to compare that clustering to using macrostates coming from an MSM. We used MSMBuilder as described above to create the MSM. Results below show that using 1,000 microstates lumped into 2 macrostates in the MSM gives a poor correlation with the NMR 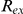, suggesting that the two inter-converting states inferred from the NMR experiments might consist of multiple metastable conformers.

#### Method 3

There is evidence that the free energy landscapes of proteins are hierarchical [29], [32]. Thus, there may be several basins even if the precision and sensitivity of the experimental data only justifies using a 2-state kinetics model [33], [34]. We attempt to bridge the gap between potential multi-state kinetics from simulation to 2-state kinetics from experiment. First, we produce an MSM with more than 2 macrostates, and cluster these macrostates into 2 clusters attempting to maximize Eq. (3). Algorithm S2 describes this procedure. To reduce the search space for the combinations of macrostates that form the minor and major states, Algorithm S2 assumes that the minor state will contain only combinations of macrostates that have at least one of the relevant H-bonds identified by Algorithm S1. We choose a number of macrostates for the MSM whose clustering into 2 exchange states gives a sufficiently high correlation according to Eq. (3).

### Betweenness Centrality Analysis of MSM

We constructed a coarser representation of the kinetics in the network using applied graph theory. Betweenness centrality (*BC*) measures the presence of a node or an edge in the shortest paths between pairs of nodes of a weighted graph [35]. It is defined as:
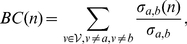
(5)where 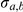 denotes the number of shortest paths of the weighted graph between nodes 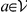 and 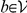 and 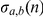 denotes the number of shortest paths where node 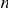 can be found. Following a similar criterion we can compute the betweenness centrality measure for all the edges in a network.

To convert the MSM to a weighted graph amenable to computing shortest paths, we transform 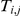, the transition probability among MSM states 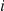 and 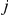, to a “free energy,” or 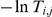. For a fixed length path between 2 macrostates, the minimum free energy path will minimize the sum of its edges. This assumes that the residency time in each metastable state is comparable, which is one of the goals of the MSM building procedure, although in practice one could also include residency times in estimating shortest paths. Thus, the problem of finding the minimum free energy or most probable paths is reduced to that of computing shortest paths. We computed 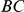 for all the edges in the MSM and reconstructed a dynamic “backbone” network of the most relevant pathways by greedily adding the edges with highest betweenness. These edges are visited by the largest number of distinct highly-probable trajectories among pairs of macrostates. The algorithm stopped once a connected network with all 40 macrostate nodes is produced.

Recent work [17] has applied Transition Path Theory (TPT) [36] for the computation of the folding flux, defined as the net flux of folding trajectories leaving the unfolded and entering the folded set, and also allows identification of pathways that are most kinetically relevant. This is an alternative method to our betweenness centrality analysis to identify a dynamic “backbone” network.

### Correlated Motions

To identify correlated motions beyond those that were studied by NMR relaxation, we use the “MutInf” method [31] to quantify correlations between residues' conformations from equilibrium simulations. Briefly, this method calculates the mutual information between pairs of residues using backbone and side chain torsions and applies statistical corrections and tests of significance for the mutual information values. It then clusters the matrix of mutual information between residues to identify groups of residues showing similar patterns of correlations. We followed the same protocol as the previously published method [31], with modifications described in the Text S1. Most notably, we filtered out snapshots in which the WW domain's heavy atoms were within 5Å of those a periodic image. This was needed because our simulation box was rather small.

## Results

### Exchange State Identification

#### Hydrogen bonding rearrangements are important descriptors of slow conformational change

We use Methods 1–3 below to identify inter-converting ensembles of structures that can provide a model of conformational dynamics consistent with the NMR exchange data. Method 1 identified major and minor exchange states with high correlation for the whole domain (protein): 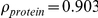 with a p-value 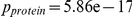 and 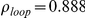 with a p-value of 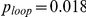 (See Figure S3 for individual residue estimation). The positive correlation suggests that H-bonds are distinguishing descriptors of the conformational states. Algorithm S1 identified unique H-bonds present within the minor conformational state (Table S3), thereby pointing to H-bond reorganization as key processes comprising the slow WW domain dynamics.

#### Simulation data is best explained by a multiple state network model

Since the experimental NMR data were fit to two-state models, we used Method 2 to construct an MSM with two macrostates. The macrostates were derived by lumping 1000 microstates. These two macrostates gave low correlation: 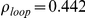 with a p-value of 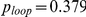 and 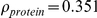 with a p-value of 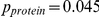 (Eq. (3)). Figure S4 compares this two-state MSM 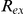 against experimental data. Thus, a two-macrostate model does not capture the full conformational plasticity of Pin1-WW.

**Figure 2:**
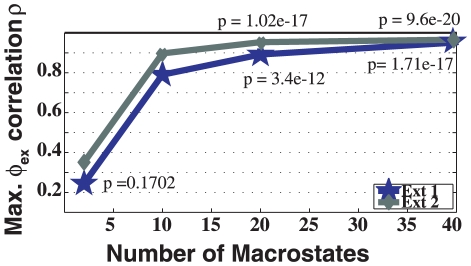
Correlation obtained by producing exchange states out of clustering different numbers of macrostates for the 1,000 microstate MSM. This plot reflects the dependence on the number of macrostates in our MSM model to achieve a maximum correlation (y-axis) and more statistically significant p-values. A MSM with 40 macrostates achieved the best partition that correlated significantly with experiment.

We tested Method 3 using the same 1000 microstates, but lumping into different numbers of macrostates. The number of macrostates that produced statistically significant results is 40 (Figures 2 and S6). Using these 40 macrostates, with an average of 37,834 conformations per macrostate, we created two exchange states. To reduce the search space needed to cluster the macrostates, the presence of H-bonds is employed by Algorithm S2 (Table S3). Macrostates with conformations containing these H-bonds were preferentially assigned to the same exchange state. This results in a minor exchange state, consisting of 2 macrostates (28 unique microstates), and a major exchange state, consisting of the remaining 38 macrostates (972 unique microstates) of the MSM.

**Figure 3:**
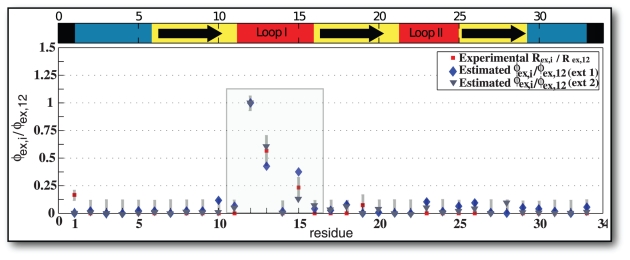
Correlation of 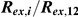 to 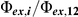 for a Markov State Model of apo Pin1-WW dynamics. For the two data sets of different lengths, Extended 1 (1 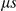) and Extended 2 (30 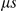), a statistically significant correlation was achieved for 40 macrostates. Bootstrapping was used to compute statistical error for the estimated 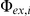, the error bars are smaller than symbol size.

Figure 3 shows the residue-specific correlation using Method 3. The correlation for the loop is 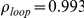 with a p-value of 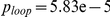, and correlation for the whole protein is 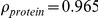 with a p-value of 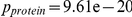. The states obtained have populations of 1,345,881 (89%) and 167,513 (11%) for the major and minor states, respectively. These populations are not too far from the experimental values of 0.7 and 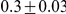. The discrepancy may be an indicator that the model estimation could benefit from longer timescale sampling: it is possible that the right major and minor states have been discovered, yet the minor state has not been sufficiently visited since the experimental timescales are longer than our simulation timescales.

We could attempt to search for the exchange states out of more macrostates in order to try to obtain better statistical agreement. However, the combinatorial complexity of the correlation maximization algorithm is the main limitation. For 40 macrostates the algorithm takes several hours while expanding the set to 60 macrostates would require months of computation. This exponential increase in complexity can be seen in Figure S7 where complexity relative to maximizing correlation for 40 macrostates is depicted. Besides, we considered that the statistical significance we achieved with 40 macrostates, with 
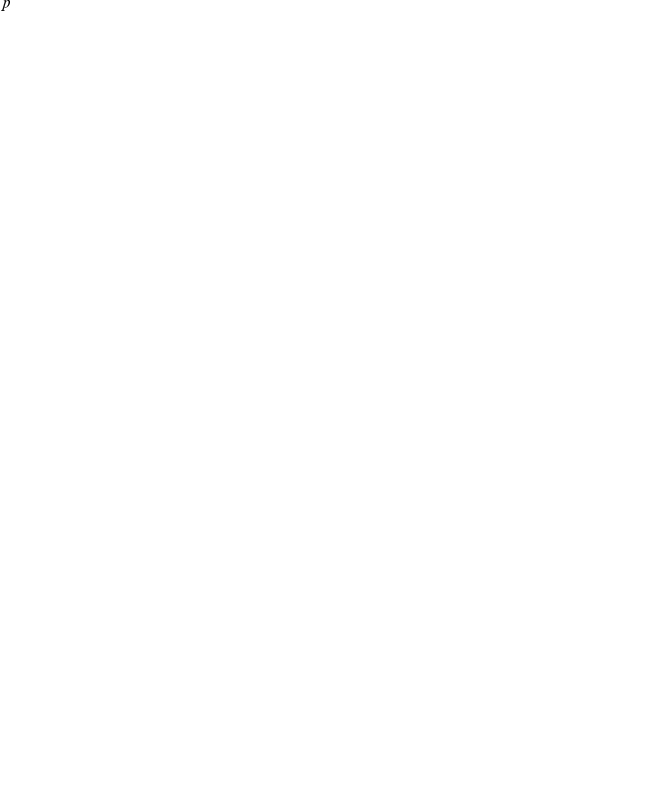
 values around 1e-20, would be only marginally improved.

**Figure 4:**
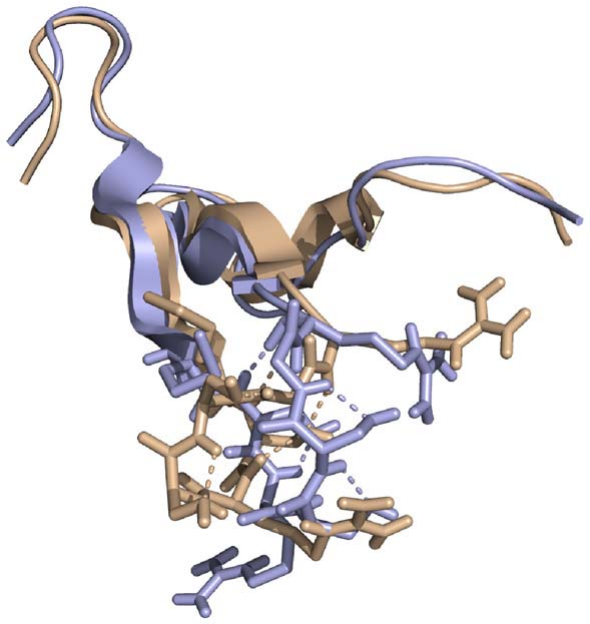
Superposition of representative structures for the two macrostates (16 and 26) belonging to the Minor State. Two different conformations of Loop 1 show a high degree of internal hydrogen bonds. The WW domain is shown in cartoon representation, with side chains in Loop 1 shown as sticks, and hydrogen bonds within Loop 1 are shown in dashes. Macrostate 16 is colored wheat and Macrostate 26 is colored light blue.

These results suggest that the hierarchical structural ensembles with inter-converting macrostates and rapidly converting microstates can explain the exchange data. Furthermore, these results stress the distinction between “states” versus “structures”. “Structures” are neighborhoods around local free energy minima (metastable or stable) in conformational space, while “states” are subsets of conformational space that may include one or more such minima, but share a common chemical feature (e.g. chemical shifts or hydrogen bond patterns). Importantly, the minor state is composed of Macrostates 16 and 26; though their Loop 1 conformations are different, they both share a high degree of internal hydrogen bonding (Figure 4). Here, the major and minor exchange states each consist of multiple macrostates. Since the macrostates represent slowly-interconverting neighborhoods of conformers, it is very useful to use a single representative structure for each macrostate.

### MSM Network Analysis

**Figure 5:**
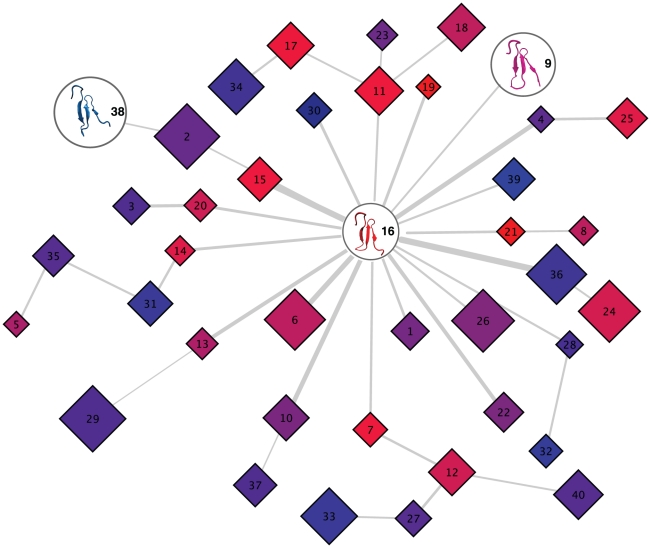
Betweenness centrality based kinetic network for the simulation ensemble Extended 2. In this kinetic network nodes represent macrostates, edge widths are proportional to their betweenness measure 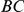. If an edge is thicker then it means that this edge belongs to several shortest paths among pairs of macrostates. Node size depends on the macrostate population. A node colored blue is closer, in RMSD terms, to the Apo Pin1-WW conformation and a red node is closer to the Holo Pin-WW structure. Figure created using Cytoscape [52].

Since the MSM produced is too fine-grained for human interpretation, we used the betweenness centrality analysis (see Methods) to produce a dynamic “backbone” network (most relevant pathways) for apo dynamics of Pin1-WW domain (see Figure 5). To compare with the experimental WW domain structures we computed the RMSD of each of the 40 representative macrostates with respect to apo Pin1-WW (PDB 1i6c) and holo Pin1-WW (PDB 1i8g). The structures were aligned with respect to the 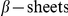 and then the Loop 1 RMSD was calculated.

#### “Invisible state” is a kinetic hub

**Figure 6:**
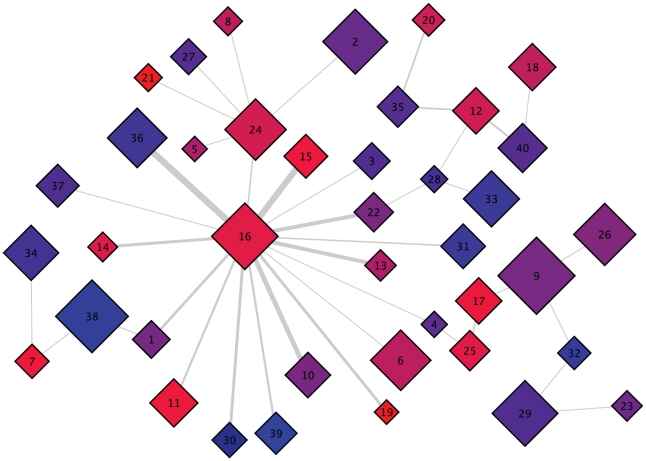
Betweeness centrality based backbone network for the simulation ensemble Extended 1. Macrostate 16 remains as the kinetic hub and states 38 and 9 are also conserved as the states with larger populations. Edge weights are proportional to betweenness centrality and node size is proportional to population. A node colored blue is closer, in RMSD terms, to the Apo Pin1-WW conformation and a red node is closer to the Holo Pin-WW structure.

Figure 5 uses a metric that ranges between −1 and 1 to color code the macrostates (the network “nodes”), where −1 and blue indicates a very apo-like structure and 1 and red a very holo-like structure (see Text S1 for metric definition and Table S1 for RMSD input values). Structural intermediate nodes are purple. Three key nodes emerged: (i) Macrostate 9, with a representative structure that is structurally an intermediate between holo and apo, is the most populated macrostate (32.7%); (ii) Macrostate 38, which is apo-like and the second most populated macrostate (20.1%); and Macrostate 16, which is holo-like, and though only moderately populated (6.6%), is the “kinetic hub”, i.e. the most central node in terms of betweenness of this kinetic network. This means that transition pathways from any macrostate to another will visit Macrostate 16 with highest probability. Macrostates 9 and 38 are also two attractors in the stationary distribution of both the long and the undersampled MSM transition matrix (see Text S1). Video S1 and Figure S5 show representative structures and populations for each macrostate. Key backbone and sidechain dihedral values of the most representative structures for each macrostate are found in Text S1. We also generated a dynamic “backbone” network using the undersampled “Ensemble 1”. Figure 6 shows this spanning tree containing 40 macrostates where node sizes are proportional to the state populations and the width of links are proportional to the betweenness centrality measure. Critically, the roles of Macrostates 16, 9, and 38 are maintained in MSM coming from different ensembles, providing further evidence of the robustness of our results.

### An Ensemble of MD Trajectories and Markov State Models Enable Exploration of Long Timescales

Implicit in the results showing agreement between the 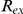 computational estimator and the experimental NMR data is that we have sampled enough conformational space. One approach to studying kinetic events in long timescales is to generate one or few very long trajectories. This approach is not feasible for millisecond simulations, unless tremendous investments on software and hardware are made. Serial simulations of this sort “waste” a lot of time waiting for rare events. Often the cause is the presence of metastability, or long-lived states. An alternative, statistical or ensemble approach is to generate an ensemble of events in parallel. This has been exploited for modeling two-state protein folding in methods such as transition path sampling and in Folding@Home. These methods are generally applicable only to two-state systems and require simulations of an unknown minimum length. Markov State Models (MSM) allow multiple states and efficient model of any system exhibiting metastability. Sampling initiated from several metastable states allows breaking up the problem of constructing the network of interconverting states. Figure 4 in [37] quantitatively illustrates the advantage of using many shorter simulations rather than few longer simulations. Often, functionally important states are also kinetically important. This has recently been found in protein folding simulations, where the native state is a “kinetic hub” [38]. This is also the case in our present study, where the putative “invisible state” is a kinetic hub. An implication of the presence of kinetic hubs in the underlying kinetic network is that one requires shorter simulations to be able to map the MSM.

Our protocol to build a kinetic model benefits from these insights: it shoots simulations out of multiple metastable states to parallelize the model construction, and uses many shorter simulations rather than fewer longer simulations. A second source of efficiency, seen as the ability to interpret events happening at time scales longer than the total amount of sampling, comes from the MSM itself. Once an MSM has been validated, it provides a model that allows extrapolation to time scales longer than those used to construct it. The explanation is that once Markovian behavior has been reached, the kinetics have a simpler form than the original molecular dynamics simulation.

To show that our approach enables extrapolation to longer timescales, we compared the MSM state populations from 2 simulation subsets of different length. A well known measure to compare two probability mass functions is the Kullback-Leibler divergence or relative entropy [39] which is defined as:
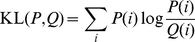
(6)


Although not symmetric, this quantity measures the extra information needed to represent (or encode) one distribution by using samples from the second distribution. A value of zero is representative of identical distributions and distinct distributions will always have increasing positive values. We used this metric to compare the population distributions of MSM's for both *Extended 1* and *Extended 2* datasets. We also compared values of these populations against a *noise* set of randomly distributed populations (Gaussian). The results are shown in Table 1. We observe values that are much closer to zero when comparing the probability mass functions of *Extended 1* and *Extended 2* than when comparing either to the *noise* mass function. This provides support of the robustness of the initial *Extended 1* dataset and the results derived from these data.

**Table 1:** Kullback-Leibler divergence between macrostates populations for the Extended 1 and Extended 2 simulation ensembles.

Kullback-Leibler Divergence	Value
KL(Ext1,Ext2)	0.02
KL(Ext2,Ext1)	0.02
KL(Ext1,noise)	0.60
KL(noise,Ext1)	0.82
KL(Ext2,noise)	0.64
KL(noise,Ext2)	0.97

### Representative Structures from MSM Macrostates Have Similar NOE Violations to Full MD Ensemble

As an independent validation of the MSM model, we compared the population-weighted “ensemble” of MSM macrostates to NOE distance restraints for PDB 1i6c (Biological Magnetic Resonance Data Bank [40] ID 4882, see Text S1 for more details). This MSM “ensemble” is reasonably consistent with NOE restraints for PDB 1i6c for residues 1–29, with less than 3% of violations over 2Å (Table 2). These NOE violations are typical of studies where molecular dynamics simulations are compared to NOE distance restraints [41]. As these distributions of NOE violations are typical of molecular dynamics simulations started from NMR structures [41]–[43], these results in combination with the agreement with the relaxation data suggest that the MSM ensemble of 40 macrostates serves as a reasonable proxy for the full ensemble. Thus, we can reasonably approximate our full ensemble with the far more human-accessible and interpretable set of 40 representative macrostate structures (see Video S1).

**Table 2:** NOE violations for MSM Ensemble.

Range	No. of violations	Percent
No violations	300	79.16
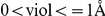	51	14.01
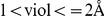	18	4.75
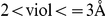	7	1.85
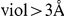	3	0.79

### Correlated Motions

Correlated protein motions are of great interest as a possible mechanism for intra-protein communication [44], [45]. Here, the NMR studies examined the 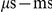 motions of backbone NHs of Loop 1. The NH motions are only a subset of the Loop 1 degrees of freedom. Thus, while the NMR data may reflect correlated motion, it may not supply enough information for their characterization. Computational approaches can bridge these information gaps. Accordingly, we investigated the possibility of correlated motions between the Loop 1 residues and other residues that would be invisible to the NMR experiments focused on 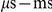 motions. We used a previously reported mutual information method, “MutInf”, to look for statistically significant correlated torsional motions in an unbiased way, independently of the MSM analysis. This entailed generating a conformational ensemble of the apo Pin1-WW domain via molecular dynamics simulations, and then identifying pairs of residues showing statistically significant correlated motions (see Methods and Text S1). Critically, this approach: (i) makes no quasi-harmonic assumptions about motions relative to an “average” structure; (ii) filters out insignificant correlations; (iii) and quantifies correlated motions in thermodynamic units. Additionally, we applied our approach to calculate the mutual information between Pin1-WW domain's 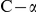 Cartesian coordinates.

#### Substrate binding in Pin1 WW results in information relay from Loop 1 to the catalytic site of Pin1 via domain interface residues in Loop 2

**Figure 7:**
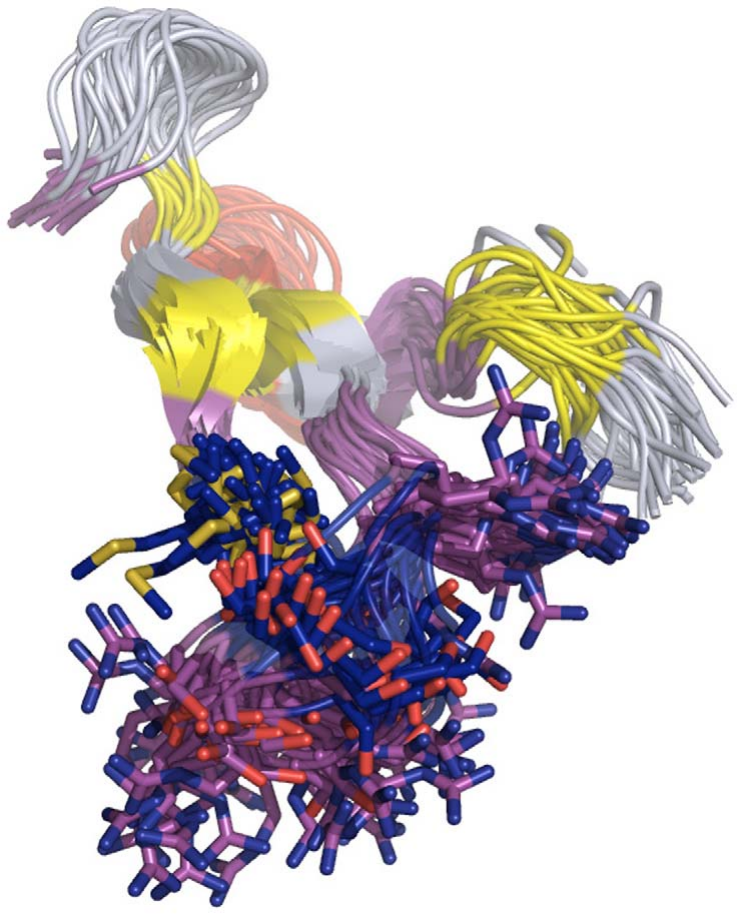
Superposition of representative structures for all 40 macrostates shows diverse conformations of Loop 1. The WW domain is shown in cartoon representation, with side chains in Loop 1 shown as sticks. Residues are colored in the same fashion as in Figure 1, i.e. according to cluster membership in the MutInf analysis.

To identify groups of residues showing similar magnitudes of correlation with other residues, we hierarchically clustered our matrix of mutual information between residues' torsions (see Methods and Text S1). The cluster with the strongest correlated motions (shown in red in Figure 1B) consists chiefly of Loop 2 residues. In full-length Pin1, these residues lie at the interface between the WW domain and its flexibly tethered isomerase domain [46]. Figure 1A further shows substantial correlation between residues in this red cluster, a blue cluster containing four residues within the substrate-binding Loop 1, a yellow cluster consisting of mostly hydrophobic core residues proximal to Loop 2, and a fourth magenta cluster containing mostly residues within Loop 1 (Figure 7). Notably, the magenta cluster contains many basic residues that form salt bridges with the phosphorylated substrate in a holo structure. Thus, substrate binding would not only perturb motions of substrate binding Loop 1, but also those of the WW-catalytic domain interface Loop 2. Focusing on the two tryptophans [47], we see that Trp29's statistically significant coupling with Trp6 does not appear to be mediated by any particular proximal shared residue (i.e. not through Gln28); rather, these two functional residues are coupled indirectly through the intervening Loop 1 (red cluster). This is most clearly seen by comparing the representative structures of macrostates 21 and 22 (Video S1). Combining these results with previous NMR studies suggests that Loop 1 can relay information about substrate binding to the catalytic site via the domain interface residues in Loop 2. We also analyzed the mutual information between 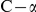 Cartesian coordinates after removing rotational/translational motions, and found the C-terminal part of Loop 2 highly correlated to the rest of the protein (Figure 1C). This Cartesian analysis complements the torsion-space analysis in Figure 1A, and is similar to previous studies [11], [48]. NMR studies implicated methyl-bearing residues in Loop 2 (Ile-23 and Thr-24 in the red cluster) in a dynamic network of residues that show perturbed dynamics upon substrate binding [49].

Other NMR studies showed coupled rotational tumbling of the two Pin1 domains in the presence but not the absence of substrate peptides of particular sequences [50]. Recently, peptides with two Pin1 binding sites separated by rigid linkers were used to ask whether Pin1 displays cooperative binding [51]. These studies found that while binding at one site facilitated binding at the other through bivalency, no significant cooperativity was observed. However, these studies did not rule out a role for substrate binding to the WW domain in substrate turnover at the active site. As correlated motions are necessary but not sufficient for allosteric crosstalk between distant sites, the functional role of this dynamic network that connects Pin1's active site to its WW-domain's substrate-binding site remains unclear and merits further study.

## Discussion

We constructed a Markov State Model (MSM) to investigate the conformational exchange dynamics detected by NMR relaxation experiments. The MSM was built from an equilibrium ensemble created from multiple simulations starting from different points of configuration space. By clustering multiple (here, 40) macrostates from a MSM into 2 exchange states, we obtained very good correlation with NMR 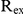 conformational exchange broadening, and reasonable agreement with NOE distance restraints.

Interestingly, the 2 macrostate MSM correlated poorly with the apparently 2-state kinetics measured by NMR. However, the hierarchical MSM (2 exchange states with 40 macrostates) correlated very well with experimental data. Thus, it is natural to hypothesize that the free energy basin of apo Pin1-WW domain is hierarchical, with several inter-converting metastable states that give rise to an apparent 2-state exchange kinetics. Such a hypothesis was enabled by the use of unrestrained simulations clustered with guidance from the NMR data. The most likely cause for masking of more subtle multi-state intrinsic kinetics is the limited number of observables within the Pin1-WW NMR relaxation studies. Ideally, additional relaxation studies at different static field strengths, temperatures, ligand concentrations, or relaxation on other nuclei might reveal the inadequacy of the two-state fits. But acquiring this wealth of data can be prohibitive for biomolecules of limited concentration or stability. And even if all such spectra were acquired, detecting greater than two exchange state can be difficult if the exchange rate constants are of similar magnitude. These considerations underscore the need for complementary computational approaches, such as proposed here.

It is possible that the main metastable states have been identified by our methodology and yet equilibrium has not been reached. This could explain the difference in populations of the major and minor states measured experimentally and computationally. In other words, the simulation has discovered the minor state but has not been able to visit it as often as the experiment due to the long timescales. It is even possible that simulation has not really discovered the invisible state measured by NMR. However, the results presented here suggest a more complex kinetic picture, where the 2 state kinetics of NMR really consists of a hierarchy of different states. Possible improvements to the methodology, besides using other NMR data when available, include even longer simulations, as well as the use of estimated kinetic rates to improve the estimator for the 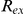. The use of adaptive sampling to construct the MSM could also improve the estimation of the transition probabilities and hence the accuracy of the kinetic agreement.

Our study focused on a two-state clustering to best correlate with the two-state analysis used in the NMR dynamics study. Of course, the potential of this computation approach is to attack other situations where a two-state model is a priori suspect. If the computational analysis implies that more than two states are at play, this can suggest additional NMR experiments that might better expose the more complicated kinetic landscape.

Analysis of the betweenness centrality of the MSM transition matrix revealed the existence of a state that is visited by most conformational transitions between metastable states. It is fascinating that this “kinetic hub” (Macrostate 16) is observed in backbone networks of both the undersampled MSM (transition matrix in Dataset S1) and the MSM with the complete data added to the model (transition matrix in Dataset S2). This provides evidence of the relevance of macrostate 16. Structural analysis of the macrostates indicate that there are apo-like states, intermediate-states, and holo-like states. This suggests that the intrinsic dynamics of apo Pin1-WW reflect a pre-existing conformational equilibrium among different functional states. Our novel interpretation of NMR relaxation data using molecular dynamics simulations and Markov State Model clustering affords an accessible model of slower Pin1 WW dynamics that is consistent with available NMR data, and intermediate between a two-conformation model and a more detailed “ensemble” of the 1000 microstates or all of the simulation snapshots. Notably, the model goes beyond a simple list of representative structures to provide a network (graph) model.

To study correlated motions beyond those examined in the MSMs or even detectable by current NMR techniques, we looked globally at correlated motions using a mutual information approach. We find that Loop 1 motions are correlated with motions involving a cluster of WW residues that predominantly lie at the interface with the catalytic domain of Pin1. These correlated motions connect Loop 1 with a previously-identified dynamic network coupling Pin1's WW domain and catalytic site.

A chief motivation for collecting more dynamic NMR data is to disclose networks of inter-converting conformations relevant for function. But such disclosure requires the appropriate computational tools. Our approach addresses this need. Moreover, it sets the stage for a more detailed understanding of other dynamic NMR parameters beyond the isotropic chemical shift, such as the residual dipolar couplings (RDCs) and residual chemical shift anisotropies (RCSAs) [4].

Our work represents a step towards building mechanistic models of intrinsic conformational dynamics by combining NMR relaxation experiments characterizing slow dynamics and Markov State Models, while simultaneously identifying correlated motions not currently observable by NMR.

## Supporting Information

Algorithm S1 (H-bond based) Exchange State Identification Algorithm.
(0.07 MB PDF)

Algorithm S2 (Hybrid H-bond and MSM based) Exchange State Identification Algorithm.
(0.12 MB PDF)

Dataset S1 The MSM transition matrix for the Extended 1 dataset.
(0.05 MB XLS)

Dataset S2 The MSM transition matrix for the Extended 2 dataset.
(0.06 MB XLS)

Figure S1 Implied time scales for the MSM macrostates. The figure shows the slowest time scale (top envelope) and the fourth slowest time scale (bottom envelope). Bootstrapping was used to compute error bars: the initial trajectory was split into 10 different pieces to allow random re-sampling with replacement.
(0.25 MB EPS)

Figure S2 Stationary distribution π of the transition probability matrix T.
(0.57 MB EPS)

Figure S3 R_ex_ estimation for WW residues using the H-bond based Method 1 versus experimental R_ex_/R_ex_(12).
(0.02 MB EPS)

Figure S4 R_ex_ estimation for WW residues for a 2 state Markov State Model versus experimental R_ex_/R_ex_(12) and the 40 state MSM using Extended 2 data set.
(0.02 MB EPS)

Figure S5 Representative structures from 40 macrostates, with the side chain of Arg-12 shown. The state population is indicated along with the macrostate index. These structures were selected using the microstate within each macrostate with the densest population, i.e. the most probable microstate. Macrostate 16, found to be a kinetic hub is shown in orange.
(0.38 MB EPS)

Figure S6 Correlation of R_ex_ estimation for different number of macrostates and using different simulation datasets.
(0.01 MB EPS)

Figure S7 Complexity of the correlation maximization algorithm for different number of MSM macrostates relative to the complexity of maximizing correlation for 40 macrostates.
(0.24 MB EPS)

Table S1 Loop and whole protein RMSD values of representative macrostate structures with respect to APO and HOLO experimental structures.
(0.03 MB PDF)

Table S2 Hydrogen bonds present in the minor state, according to Exchange State Identification Method 1. Atom names according to CHARMM 27 force field.
(0.04 MB PDF)

Table S3 Dihedral values for Arg-12, Ser-13, and Gly-15 of representative structures for each macrostate of the MSM.
(0.03 MB PDF)

Text S1 Additional details for methods and results sections as well extra figures and tables.
(0.15 MB PDF)

Video S1 This movie depicts the 3-D structures of each of the representative conformations of the Markov State Model of Pin1 WW domain.
(3.75 MB MOV)
