# Supplementary material for: Modeling Conformational Ensembles of Slow Functional Motions in Pin1-WW
Source: PLoS Comput Biol. 2010 Dec 2;6(12):e1001015. doi: 10.1371/journal.pcbi.1001015 (PMC2996313; doi:10.1371/journal.pcbi.1001015)
Supplement: Algorithm S1 — (H-bond based) Exchange State Identification Algorithm. (0.07 MB PDF) [file pcbi.1001015.s001.pdf]

Algorithm S1. (H-bond based) Exchange State Identification Algorithm

Input: WW domain trajectory frames in set $\mathcal{T}$.

while $|\rho_{best} - \rho_{old}| > \epsilon$ **do** ▷ $\epsilon = 0.0001$
  for All i H-bonds in loop with probability of occurrence > 0 **do**
    $\mathcal{N} \leftarrow \mathcal{N} \cup f(i)$ ▷ $f(i)$: set of frames with H-bond i
    if $\rho(\mathbf{R_{ex}}, \mathbf{\Phi}_{ex}(\mathcal{N}, \mathcal{T} \setminus \mathcal{N})) > \rho_{best}$ **then**
      $\rho_{old} \leftarrow \rho_{best}$
      $\rho_{best} \leftarrow \rho(\mathbf{R_{ex}}, \mathbf{\Phi}_{ex}(\mathcal{N}, \mathcal{T} \setminus \mathcal{N})$
      $\mathcal{N}_{best} \leftarrow \mathcal{N}_{best} \cup i$
      $\mathcal{N} \leftarrow \mathcal{N}_{best}$
    end if
  end for
end while
Output: $\mathcal{A} \leftarrow \mathcal{N}_{best}$ and $\mathcal{B} \leftarrow \mathcal{T} \setminus \mathcal{N}_{best}$
