# Supplementary material for: Modeling Conformational Ensembles of Slow Functional Motions in Pin1-WW
Source: PLoS Comput Biol. 2010 Dec 2;6(12):e1001015. doi: 10.1371/journal.pcbi.1001015 (PMC2996313; doi:10.1371/journal.pcbi.1001015)
Supplement: Algorithm S2 — (Hybrid H-bond and MSM based) Exchange State Identification Algorithm. (0.12 MB PDF) [file pcbi.1001015.s002.pdf]

**Algorithm S2. (Hybrid H-bond and MSM based) Exchange State Identification Algorithm**

**Input:**
Chemical Shift Values per residue. $CS = \{CS_1, CS_2, \ldots, CS_{33}\}$ where $CS_i \in \mathcal{R}^N$
where $N$ is the total number of frames in the simulation.
Macro states from MSMBuilder. $MS = \{MS_1, MS_2, \ldots, MS_M\}$
where $M$ is the total number of macrostates.
H-bond frames in minor state from Algorithm S1. $HB = \{HB_1, HB_2, \ldots, HB_H\}$
where $H$ is the total number of relevant H-bonds found by Algorithm S1.

```
function SuperStateSearch(CS,MS,HB)
    SC ← ∅
    for i ← 1...M do                                     ▷ M = 40 in this study
        if MS_i ∩ HB > δ then        ▷ δ is the minimum intersection fraction δ = 0.002
            SC ← SC ∪ MS_i           ▷ SC Identifies relevant macrostates to reduce search
        end if
    end for
    for k ← 1...⌊|SC|/2⌋ + 1 do
        for z ← 1...(|SC| choose k) do
            StatesA ← SC(z)          ▷ SC(x) is one of (|SC| choose k) macrostate combinations in SC
            StatesB ← {MS_1, MS_2, ...MS_40} \ StatesA
            Φ_ex ← EstimateΦ_ex(StatesA, StatesB, CS)     ▷ See Equation (2) in main paper
            ρ_new ← ρ(Φ_ex, R_ex)
            if ρ_new > ρ_best then
                ρ_best ← ρ_new
                SuperA ← StatesA
                SuperB ← StatesB
            end if
        end for
    end for
    return SuperA, SuperB, ρ_best
end function
```
