# Supplementary material for: Modeling Conformational Ensembles of Slow Functional Motions in Pin1-WW
Source: PLoS Comput Biol. 2010 Dec 2;6(12):e1001015. doi: 10.1371/journal.pcbi.1001015 (PMC2996313; doi:10.1371/journal.pcbi.1001015)
Supplement: Table S1 — Loop and whole protein RMSD values of representative macrostate structures with respect to APO and HOLO experimental structures. (0.03 MB PDF) [file pcbi.1001015.s012.pdf]

Table S1. Loop and whole protein RMSD values of representative macrostate structures with respect to APO and HOLO experimental structures

Macrostate	RMSD APO protein	RMSD APO loop	RMSD HOLO protein	RMSD HOLO loop
1	7.07	4.15	6.85	4.14
2	6.66	4.23	6.49	4.17
3	6.95	4.21	6.92	4.16
4	7.25	4.21	7.25	4.31
5	6.91	4.57	6.65	3.75
6	6.76	4.19	6.30	3.69
7	7.22	4.34	6.84	4.11
8	6.81	4.26	6.48	3.73
9	6.87	3.95	6.76	4.50
10	6.45	3.88	6.13	4.27
11	6.83	4.69	6.41	3.29
12	6.74	4.33	6.40	3.33
13	6.73	4.58	6.46	3.39
14	6.75	4.82	6.41	3.33
15	6.75	4.71	6.37	3.37
16	6.78	4.74	6.49	3.35
17	6.61	4.82	6.23	3.37
18	6.82	4.41	6.55	3.40
19	6.85	4.88	6.28	3.69
20	6.68	4.21	6.26	3.75
21	7.25	4.00	6.81	3.82
22	6.53	4.04	6.27	3.63
23	6.10	4.30	6.10	4.02
24	6.55	4.40	6.29	3.35
25	6.73	4.08	6.47	3.35
26	6.30	3.49	6.30	3.99
27	6.29	3.39	6.47	4.39
28	5.98	3.34	5.89	4.13
29	6.12	3.43	6.13	4.62
30	6.10	3.50	6.23	4.24
31	6.45	3.53	6.64	4.33
32	6.40	3.36	6.62	4.47
33	6.35	3.55	6.59	4.40
34	6.32	3.77	6.32	3.89
35	6.06	3.65	5.83	4.98
36	5.72	3.54	5.56	4.37
37	6.42	3.57	6.38	4.17
38	6.55	3.40	6.66	4.18
39	6.10	3.78	6.16	4.53
40	6.17	3.99	6.26	4.18
