# Supplementary material for: Modeling Conformational Ensembles of Slow Functional Motions in Pin1-WW
Source: PLoS Comput Biol. 2010 Dec 2;6(12):e1001015. doi: 10.1371/journal.pcbi.1001015 (PMC2996313; doi:10.1371/journal.pcbi.1001015)
Supplement: Table S2 — Hydrogen bonds present in the minor state, according to Exchange State Identification Method 1. Atom names according to CHARMM 27 force field. (0.04 MB PDF) [file pcbi.1001015.s013.pdf]

Table S2. Hydrogen bonds present in the minor state, according to Exchange State Identification Method 1. Atom names according to CHARMM 27 force field.

Residue pairs	*Number of H-Bonds*	Donor-Acceptor Atoms
Arg16-Ser14	(2)	HN-N-OG, HH11-NH1-OG
Arg16-Ser11	(1)	HN-N-O
Arg12-Arg12	(1)	HE-NE-O
Arg12-Arg12	(1)	HH11-NH1-O
Ser11-Ser14	(2)	HG1-OG-O, HN-N-O
Ser11-Ser13	(1)	HG1-OG-OG
