# Supplementary material for: Modeling Conformational Ensembles of Slow Functional Motions in Pin1-WW
Source: PLoS Comput Biol. 2010 Dec 2;6(12):e1001015. doi: 10.1371/journal.pcbi.1001015 (PMC2996313; doi:10.1371/journal.pcbi.1001015)
Supplement: Table S3 — Dihedral values for Arg-12, Ser-13, and Gly-15 of representative structures for each macrostate of the MSM. (0.03 MB PDF) [file pcbi.1001015.s014.pdf]

Table S3. Dihedral values for Arg-12, Ser-13, and Gly-15 of representative structures for each macrostate of the MSM.

Macrostate	phi12	psi12	chi12	phi13	psi13	chi13	phi15	psi15
1	-101.3	-62.6	178.9	-101.3	-67.9	-163.5	179.5	-170.4
2	-67.5	-53.5	168.7	-113.2	-114.1	-178.1	141.4	-131.4
3	-76.2	-52.4	178.9	-91.3	-127.2	-165.6	165.4	-162.5
4	-66.1	-68.4	175.3	-117.0	-83.8	-151.9	-178.0	-176.4
5	-96.2	-177.3	-48.7	-55.8	-44.8	-52.2	156.9	-169.8
6	-67.2	-50.5	-171.3	-133.1	-167.5	-160.5	161.9	-121.2
7	-79.8	-55.7	-148.2	-138.1	155.1	-62.3	89.0	-41.4
8	-77.2	-54.5	163.5	-143.6	-153.9	-173.1	129.5	-104.8
9	-110.1	-72.3	-170.1	-71.6	150.2	-170.2	-175.3	-103.5
10	-85.0	-64.8	174.1	-76.0	154.2	-160.2	170.0	-83.7
11	-108.5	-50.1	-63.8	-152.9	-57.7	-179.7	159.9	-114.9
12	-87.4	-120.0	-62.1	-99.1	-47.0	-171.8	104.6	-95.4
13	-105.0	-121.5	-63.2	-83.8	-38.6	-58.5	123.5	-121.5
14	-111.7	-117.0	-46.1	-72.8	-49.1	-57.8	111.0	-113.7
15	-81.4	-151.0	-47.7	-69.3	-28.8	53.2	128.6	-93.8
16	-104.6	-125.1	-59.5	-70.5	-55.0	70.7	106.3	-154.8
17	-96.4	-107.2	-52.4	-76.2	-70.7	43.2	116.1	-126.9
18	-100.8	-125.3	-52.9	-82.1	-44.0	-49.8	106.3	-137.1
19	-93.9	-169.2	-70.7	-61.6	-61.9	-26.9	100.0	-35.3
20	-97.4	-99.9	-65.5	-84.1	-60.6	56.9	121.1	-105.1
21	-90.6	-89.2	-58.2	-85.6	-165.1	37.5	159.2	-76.1
22	65.5	-115.1	-56.3	-70.6	-54.6	62.9	166.5	-89.8
23	72.8	-89.6	-161.9	-123.3	-83.8	69.7	148.8	-178.0
24	68.9	-132.5	-154.8	-83.8	-72.8	72.3	123.3	-69.8
25	80.7	-92.8	-158.5	-164.0	-40.0	65.0	-135.3	-32.9
26	-84.7	-61.4	178.6	-88.6	-69.6	57.7	-168.7	63.5
27	-84.8	2.3	-157.1	-74.8	-124.6	51.9	-137.7	64.5
28	-60.8	-59.0	-174.1	-79.8	-75.3	61.7	146.4	37.5
29	-132.2	-33.8	-179.2	-71.8	-54.3	60.2	-140.5	43.2
30	-105.6	-10.5	-162.2	-106.7	-92.7	46.5	-96.5	75.1
31	-77.2	-67.4	-60.7	-60.8	-50.5	55.5	-95.8	57.3
32	-83.7	14.7	-56.1	-87.1	-78.8	50.5	115.6	44.4
33	-64.7	-43.3	-59.5	-67.3	-25.4	65.4	92.7	31.4
34	-76.4	-68.4	-169.3	-85.5	-47.6	167.0	-103.3	36.4
35	-100.6	-22.0	-50.6	-85.6	-84.0	-176.5	-82.2	-21.5
36	-76.2	-47.1	-168.3	-96.7	-76.3	-71.8	-84.8	61.5
37	-98.9	-49.4	179.0	-74.8	-41.6	53.1	113.7	-2.0
38	-74.3	-31.0	-58.1	-81.8	-95.1	35.4	109.1	42.8
39	-105.9	-58.2	179.8	-79.5	-19.9	-53.3	116.1	-70.4
40	-84.5	-39.1	-72.8	-75.9	-29.3	-46.2	111.3	-53.7
