# Supplementary material for: Modeling Conformational Ensembles of Slow Functional Motions in Pin1-WW
Source: PLoS Comput Biol. 2010 Dec 2;6(12):e1001015. doi: 10.1371/journal.pcbi.1001015 (PMC2996313; doi:10.1371/journal.pcbi.1001015)
Supplement: Text S1 — Additional details for methods and results sections as well extra figures and tables. (0.15 MB PDF) [file pcbi.1001015.s015.pdf]

Supporting information for "Modeling conformational ensembles of slow functional motions in Pin1-WW"

Faruck Morcos[1,2], Santanu Chatterjee[1,2], Christopher L. McClendon [3], Paul R. Brenner [4], Roberto López-Rendón [5], John Zintsmaster [6], Maria Ercsey-Ravasz [1,7], Christopher R. Sweet [1,4], Matthew P. Jacobson [3], Jeffrey W. Peng [6,*], Jesús A. Izaguirre [1,2,*]
1 Interdisciplinary Center for Network Science and Applications, Notre Dame, IN, USA
2 Department of Computer Science and Engineering, University of Notre Dame, Notre Dame, IN, USA
3 Graduate Group in Biophysics and Department of Pharmaceutical Chemistry, University of California San Francisco, San Francisco, CA, 94158
4 Center for Research Computing, University of Notre Dame, Notre Dame, IN, USA
5 Facultad de Ciencias, Universidad Autónoma del Estado de México, Toluca, México
6 Department of Chemistry and Biochemistry, University of Notre Dame, Notre Dame, IN, USA
7 Department of Physics, University of Notre Dame, Notre Dame, IN, USA
∗ Corresponding authors: jpeng@nd.edu, izaguirr@nd.edu

NOE violations calculation

NOE distance restraints from Pin1-WW were used to assess the quality of the ensemble of MSM macrostate structures. Proton-proton distances were computed using AQUA [3], and then ensemble averaged (using $1/r^6$ scaling) using a python script. Only restraints for residues 1-29 were included in this analysis as the NOEs were measured with the longer construct.

NOE upper bound violations were computed over the population-weighted "ensemble" of representative structures from the MSM, as in the text. As these distributions of NOE violations are typical of molecular dynamics simulations started from NMR structures [4–6], these results in combination with the agreement with the relaxation data suggest that the MSM ensemble of 40 macrostates serves as a reasonable proxy for the full conformational ensemble.

H-bond calculation

We consider H-bonds to exist if donor and acceptor atoms are within a distance of 3.5 Å and if the bond angle is larger or equal than 150°. Tcl and VMD [7] scripts were implemented to detect the presence of Loop 1 hydrogen bonds for each conformation of the simulated trajectory.

Structural metric

This metric is defined as:

$$C(m) = \frac{\mathrm{A}(m)_{RMSD} - \mathrm{H}(m)_{RMSD}}{\max_{m\in\mathcal{V}}\mathrm{H}(m)_{RMSD} - \min_{m\in\mathcal{V}}\mathrm{A}(m)_{R}} \quad (1)$$

where $\mathrm{H}(m)_{RMSD}$ and $\mathrm{A}(m)_{RMSD}$ are the RMSD values for each macrostate m with respect to holo and apo respectively. The holo structure is model 0 of the NMR PDB 1i8g, and the apo structure is model 0 of the NMR PDB 1i6c. The latter is also the starting point of the MD simulations. A value of $C(m)$ close to -1 means that a macrostate is closer to apo and further away from holo and viceversa for $C(m)$ close to 1. Loop and whole protein RMSD values of representative macrostate structures with respect to APO and HOLO experimental structures are shown in Table S1.

Data Sampling and Model Robustness

We applied our methodology to 3 different simulation data sets of increasing sampling length (0.5, 2, and 30 μs). Figure S6 compares the original trajectory T_1, the *Extended 1* and *Extended 2* sets. This figure shows an improvement on p-value and actual correlation when adding more macrostates, and also when adding more sampling, particularly between the original simulation and *Extended 1* set used in the paper. The data from *Extended 2* improves both correlation and statistical significance of our results.

Another important point is the observation that a two-state MSM cannot achieve significant correlations with experimental R_{ex}. Figure S4 shows the detailed estimations of R_{ex} for all the residues in the WW domain. It can be seen that a poor correlation when compared with experimental data and the 40 macrostate MSM using the *Extended 2* sampling data. This performance is mainly due to the fact that relaxation dispersion is predicted to exist in regions outside the loop.

For the MSM of the *Extended 1* set we also computed a minimum spanning tree based on the betweenness centrality measure described in the paper. The key role of Macrostate 16 is retained in this dataset. Again, macrostates of higher populations have to visit Macrostate 16, a low population state, in order to visit other conformational states. This suggests an inherent robustness on the results obtained by both data sets *Extended 1* and *Extended 2* and highlights the conclusions of our findings.

Correlated Motions

As explained in the paper, we use the “MutInf” method [10] to quantify correlations between residues’ conformations from equilibrium simulations. Briefly, this method calculates the mutual information between pairs of residues, applies statistical corrections and tests of significance for the mutual information values, and then clusters the matrix of mutual information between residues to identify groups of residues showing similar patterns of correlations.

We followed the same protocol as the previously published method [10], with the following modifications. We split the APO *Extended 1* ensemble into six equal-sized segments, after removing the 10% of snapshots where the WW domain’s heavy atoms were within 5 Å of those of a periodic image. Also, we added a statistical bootstrapping approach to the protocol as an additional statistical filter to require the reproducibility of a correlation between a pair of torsions. We split the full trajectory into six time segments, and take four out of six segments at a time as a sample ensemble, or “block”, from which we aggregate the histogram counts for the two torsions and calculate the mutual information for each sample ensemble. This bootstrapping approach is similar to block-averaging; our “blocks” are composed of multiple, not-necessarily contiguous, and large time segments. The Wilcoxon signed-rank test is used to test the null hypothesis that the average (corrected) mutual information is zero against a one-sided alternative. If the p-value is less than or equal to $\alpha = 0.01$, the average of the mutual information values for the “blocks” is reported, otherwise it is zeroed.

To calculate the mutual information between each pair of residues, we take the sum of the mutual information over all pairs of ϕ, ψ, and χ dihedral angles, each pair comprising one angle from each residue [10]. We then clustered the mutual information between pairs of residues using the “heatmap” function in the R statistical package with a Euclidean distance metric.

Additionally, we calculated the mutual information between residues’ C-α coordinates using the same procedure as above, using x, y, and z coordinates in place of ϕ, ψ, and χ. Rotational and translational motion was removed prior to analysis by a rotational/translational fit involving only C-α atoms.

Video S1

Video S1. A movie with all 40 representative macrostate structures shows the diverse, slowly-interconverting Pin1-WW domain conformations. Major state and Minor state membership and macrostate number are

indicated in each frame. Residues are colored as in Figure 1 in the text, i.e. according to the MutInf cluster to which they belong.

References

1. Phillips JC, Braun R, Wang W, Gumbart J, Tajkhorshid E, Villa E, Chipot C, Skeel RD, Kale L, Schulten K (2005) Scalable molecular dynamics with NAMD. *J Comput Chem* 26:1781–1802.

2. Neal S, Nip AM, Zhang H, Wishart DS (2003) Rapid and accurate calculation of protein ^{1}H, ^{13}C and ^{15}N chemical shifts. *J Biomol NMR* 26:215–240.

3. Laskowski RA, Rullmann JAC, MacArthur MW, Kaptein R, Thornton JM (1996) AQUA and PROCHECK-NMR: Programs for checking the quality of protein structures solved by NMR. *J. Biomol. NMR* 8:477-486.

4. Kony DB, Hünenberger PH,van Gunsteren WF 2007 Molecular dynamics simulations of the native and partially folded states of ubiquitin: Influence of methanol cosolvent, pH, and temperature on the protein structure and dynamics. *Protein Science* 16(6):1101-1118.

5. Merkley ED, Bernard B, Daggett, V (2007) Conformational Changes below the Tm: Molecular Dynamics Studies of the Thermal Pretransition of Ribonuclease A. Biochemistry 47(3):880-892.

6. Zagrovic B, Gattin Z, Lau J, Huber M, van Gunsteren, W (2008) Structure and dynamics of two β-peptides in solution from molecular dynamics simulations validated against experiment. *European Biophysics Journal* 37(6):903-912.

7. Humphrey W, Dalke A, Schulten K (1996) VMD: Visual molecular dynamics. *J Mol Graph* 14:33–8, 27–8.

8. Chodera JD, Singhal N, Pande VS, Dill KA, Swope WC (2007) Automatic discovery of metastable states for the construction of Markov models of macromolecular conformational dynamics. *J Chem Phys* 126:155101.

9. Noé F, Schütte C, Vanden-Eijnden E, Reich L, Weikl TR (2009) Constructing the equilibrium ensemble of folding pathways from short off-equilibrium simulations. *Proc Natl Acad Sci U S A* 106:19011-19016.

10. McClendon CL, Friedland G, Mobley DL, Amirkhani H, Jacobson MP (2009) Quantifying Correlations Between Allosteric Sites in Thermodynamic Ensembles. *J Chem Theor Comput* 9:2486–2502

11. Jacobs DM, Saxena K, Vogtherr M, Bernadó P, Pons M, Fiebig KM (2003) Peptide binding induces large scale changes in inter-domain mobility in human Pin1. *J Biol Chem* 278:26174–26182.

12. Daum S, Lücke C, Wildemann D, Schiene-Fischer C (2007) On the Benefit of Bivalency in Peptide Ligand/Pin1 Interactions. *J Mol Biol* 374:147–161.
